# Ureteral Stent Retrieval Using the Crochet Hook Technique in Females

**DOI:** 10.1371/journal.pone.0029292

**Published:** 2012-01-03

**Authors:** Takashi Kawahara, Hiroki Ito, Hideyuki Terao, Takuya Yamagishi, Takehiko Ogawa, Hiroji Uemura, Yoshinobu Kubota, Junichi Matsuzaki

**Affiliations:** 1 Department of Urology, Ohguchi Higashi General Hospital, Yokohama, Kanagawa, Japan; 2 Department of Urology, Yokohama City University Graduate School of Medicine, Yokohama, Kanagawa, Japan; University of Colorado School of Medicine, United States of America

## Abstract

**Introduction:**

We developed a method for ureteral stent removal in female patients that requires no cystoscopy or fluoroscopic guidance using a crochet hook. In addition, we also investigated the success rate, complications and pain associated with this procedure.

**Methods:**

A total of 40 female patients (56 stents) underwent the removal of ureteral stents. All procedures were carried out with the patients either under anesthesia, conscious sedation, or analgesic suppositories as deemed appropriate for each procedure including Shock Wave Lithotripsy (SWL), Ureteroscopy (URS), Percutaneous Nephrolithotomy (PCNL), and ureteral stent removal. At the time of these procedures, fluoroscopy and/or cystoscopy were prepared, but they were not used unless we failed to successfully remove the ureteral stent using the crochet hook. In addition, matched controls (comprising 50 stents) which were removed by standard ureteral stent removal using cystoscopy were used for comparison purposes.

**Results:**

A total of 47 of the 56 stents (83.9%) were successfully removed. In addition, 47 of 52 (90.4%) were successfully removed except for two migrated stents and two heavily encrusted stents which could not be removed using cystoscopy. Ureteral stent removal using the crochet hook technique was unsuccessful in nine patients, including two encrustations and two migrations. Concerning pain, ureteral stent removal using the crochet hook technique showed a lower visual analogue pain scale (VAPS) score than for the standard technique using cystoscopy.

**Conclusions:**

Ureteral stent removal using a crochet hook is considered to be easy, safe, and cost effective. This technique is also easy to learn and is therefore considered to be suitable for use on an outpatient basis.

## Introduction

Ureteral stents were first reported by Zimskind *et al.* in 1967. Thereafter, ureteral stents were essential for maintaining ureteral patency in the management of various benign and malignant forms for ureteral obstruction. Serious complications, including migration, fragmentation, and stone formation still occur, especially when stents have been forgotten for a long time [1], [2], [3], [4]. The incidence of encrustation increases with the duration that the stent remains indwelling [5]. Therefore, every 6 weeks to 6 months either stent exchange or removal is necessary [1], [2], [6], [7], [8], [9], [10].

Ureteral stent removal is usually performed under cystoscopy. On the other hand, fluoroscopic guidance of ureteral stent removal with a snare loop or foreign body retrieval forceps has also been reported [11], [12], [13], [14]. We investigated the ureteral stent removal technique using a crochet hook without cystoscopy or fluoroscopic guidance in female patients, and also investigated the success rate, complications and pain in comparison to the standard technique of cystoscopy.

## Methods

A total of 40 female patients (56 ureteral stents) underwent the removal of ureteral stents using a crochet hook. Cystoscopy confirmed stent migration to the ureter in two patients. Two of these removed stents also needed additional therapy by means of Shock Wave Lithotripsy (SWL) and ureteroscopy (URS). The patient characteristics are shown in Table 1. Informed consent was obtained from all patients prior to participation in this study. The stents were 5 to 8Fr with a loop or double pigtails configuration (Table 1). Twenty-one stents were inserted after initial URS, 7 stents for pre-stenting before URS, 13 stets for eliminating hydronephrosis, 1 stent for pain relief, 9 stents for urosepsis and 5 stents for conclusion of URS or percutaneous nephrolithotomy (PCNL). Matched controls (comprising 50 stents) with the same backgrounds regarding age, side, type of stent, indications for stenting and anesthesia, were used for comparison purposes.

**Table 1:** Patient Characteristics.

Variables	Number (%) or Median (mean ± SD)		*P*
	CH Removal	STD Removal	
No. of stents	56	50	
No. of Pts.	40	42	
Age (yr)	64 (62.5±16.2))	58.8 (58.1±15.3)	n.s.
Indication for stenting			
Stone disease (%)	56 (100%)	50 (100%)	n.s.
Anesthesia/Sedation/Pain Killer			
General anesthesia	33 (58.9%)	26 (52.0%)	n.s.
General and epidural anesthesia	3 (5.4%)	2 (4.0%)	
Spinal anesthesia	1 (1.8%)	2 (4.0%)	
Conscious Sedetion	10 (17.6%)	9 (18.0%)	
NSAIDs suppo.	9 (16.1%)	10 (20.0%)	
Side			
Right (%)	28 (50.0%)	26 (52.0%)	n.s.
Left (%)	28 (50.0%)	24 (48.0%)	
Type of Stent			
Loop (%)	47 (83.9%)	41 (82.0%)	n.s.
Double Pigtails (%)	9 (16.1%)	9 (18.0%)	

NSAIDs: non steroidal anti infllamatory drugs.

**Figure 1:**
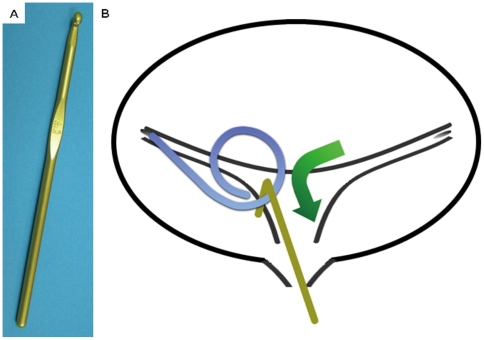
Crochet hook and the crochet hook technique. a: Crochet hook. b: The hook was inserted into the bladder and used to draw out the distal end of the ureteral stent. (arrow) The distal end of the ureteral stent is grasped with the crochet hook and pulled out through the urethra.

**Figure 2:**
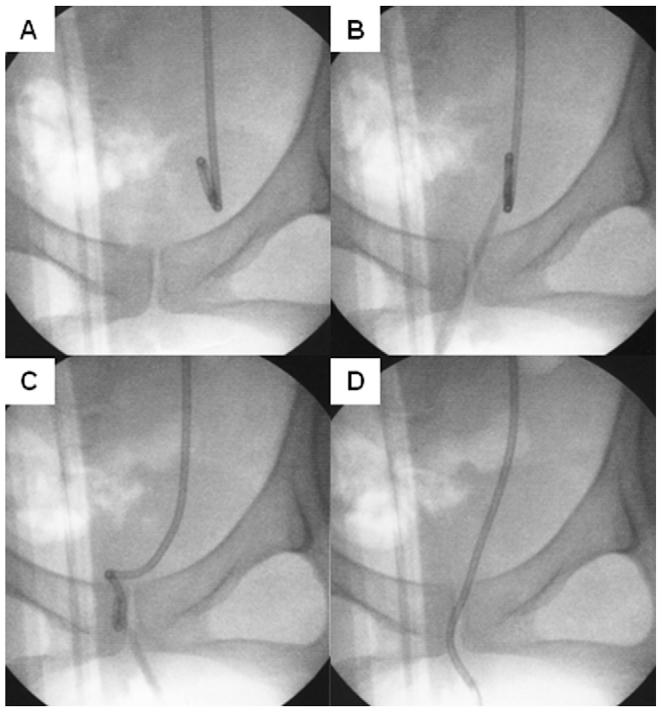
The images of this procedure. These images show the crochet hook technique. However we did not use fluoroscopy in this study.

A crochet hook made of metal was selected and was sterilized by autoclaving. (Fig. 1a) A lidocaine gel (2%) was spread on the hook, and the crochet hook was inserted into the urethra. The crochet hook was advanced toward ureteral orifice, and it was carefully passed from the ureteral orifice to the urethra and passed softly over the bladder mucosa (Fig. 1b). Figure 2 shows an image of this procedure; however, we did not use fluoroscopy in this study. The surgeon repeated the same procedure up to 5–10 times until the distal end of the stent passed from the external urethral orifice, or patient's complaint of pain. The ureteral stent was removed using fluoroscopic guidance or cystoscopically if it could not be withdrawn using the crochet hook.

All procedures were carried out with the patients under anesthesia, conscious sedation, or analgesic suppositories, depending upon the procedure. A total of 36 patients were under general anesthesia with or without epidural anesthesia during URS and PCNL. One patient was under spinal anesthesia during URS. Ten patients received SWL under conscious sedation, while nine patients who underwent outpatient ureteral stent removal only received a diclofenac sodium (50 mg) suppository. All patients were treated in the lithotomy position., both fluoroscopy and cystoscopy were prepared for URS and PCNL and cystoscopy was prepared for SWL and ureteral stent removal at the time of procedures, but not used for ureteral stent removal unless we failed to perform the stent removal using a crochet hook.

The bladder mucosa was observed at the time of ureteral stent insertion at conclusion of the operation (URS and PCNL) in 37 patients. At the end of the procedure, a bladder catheter was inserted for URS and PCNL, which was removed the day after each procedure. Patients were given antibiotics following each procedure in the usual fashion.

The correlation between stent position and rate of success was investigated in two groups. Ho CH *et al*. described the ureteral position into 3 groups: [15] (1) a short stent: with either pigtail not curled completely. (2) an appropriate stent: with the intravesical pigtail not across the midline (pubic symphysis) and the intrarenal pigtail in the middle portion of the kidney shadow, and (3) an overlong stent: with the intravesical pigtail across the midline. The loop type stents were divided into three groups: (1) a short stent: distal loop position closed less than 1 cm in the vesicle. (2) an appropriate stent: distal loop position open more than 1 cm and less than 5 cm in the vesicle. (3) an overlong stent: distal loop position more than 5 cm in the vesicle. We assigned the procedures into two groups using these three groups: 1) good position, including an appropriate stent and an overlong stent and 2) poor position including a short stent.

A visual analog pain scale (VAPS) was used to assess pain in comparison to cystoscopy in 12 stents using the crochet hook technique and 10 stents using the standard technique. Outpatients treated with cystoscopy and those treated with a crochet hook for stent removal, were asked to grade the pain level experienced by completing a 5-scale validated VAPS after each procedure.

### Statistical analysis

All continuous variables are expressed as the mean ± SD. The numerical data were compared by Student's *t*-test. A *p*-value of 0.05 or less was considered to be significant.

## Results

Since November 2010 to February 2011, 40 patients (56 stents) underwent ureteral stent removal using the crochet technique and 42 patients (50 stents) underwent ureteral stent removal using the standard technique by means of cystoscopy. The patient data, stent data, the number of sessions, complications, and clinical success are listed in Table 1. All patients suffered from ureteral stones. Ureteral stent removal was unsuccessful with additional procedure of fluoroscopy and cystoscopy in four patients with two encrustation and two migrations. Two stents migrated to the ureter; however, no additional procedure was required because of scheduled URS.

Forty-seven of 56 stents were successfully removed (83.9%). In addition, 47 of 52 (90.4%) were successfully removed except for two migrated stents and two heavily encrusted stents which could not be removed using cystoscopy. One case of ureteral stent encrustation was needed to perform ureteroscopy with Holmium: yttrium aluminum garnet (Ho: YAG) laser for removal of ureteral stent. However, for the other it was necessary to perform SWL for removal. (Table 2)

**Table 2:** Summary of retrieval ureteral stent using crochet technique.

Variables	Number (%) or Median (mean ± SD)		*P*
	CH Removal	STD Removal	
No. of Stents	56	50	n.s.
No. of sessions	3 (2.8±1.9)	1 (1.1±0.3)	<0.001
Success of remval	47/56 (83.9%)	46/50 (92.0%)	n.s.
Reason of irremovable			
Encrustation	2 (3.6%)	2 (4.0%)	n.s.
Migration	2 (3.6%)	2 (4.0%)	n.s.
Complications			
Active bleedings	0 (0%)	0 (0%)	n.s.
Hematuria	30 (53.6%)	27 (54.0%)	n.s.
Urinary infection	1 (1.8%)	2 (4.0%)	n.s.
Needed of additional procedure			
Cystoscopy	4 (7.1%)	-	
Fluoroscopy	1 (1.8%)	-	

VAPS: 5 grades visual analogue pain scale.

**Figure 3:**
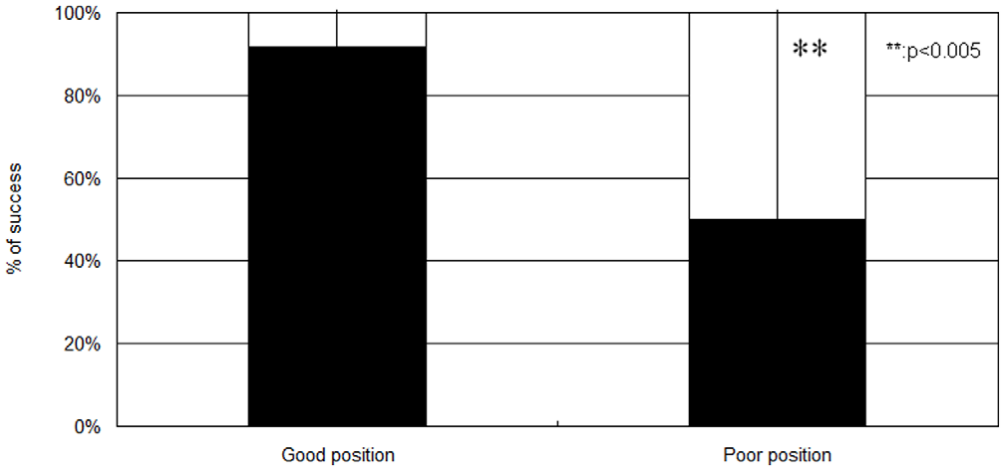
A comparison of the success rate between the good and poor ureteral stent position groups. The success rate of ureteral stent removal was significantly higher for stents in a good stent position than in a poor position. (p<0.001).

The technical success rate was significantly higher in a good position (91.7%) in comparison to the poor stent position group (50.0%). (Fig. 3) Irremovable stents in the poor position group were removed either cystoscopically or with fluoroscopy assisted a crochet hook.

Minor complications, including gross hematuria occurred in most of the patients (31 stents: 57.4%), which were also complications of URS and PCNL. No active bleedings and from the bladder mucosa were observed after URS and PCNL. None of the outpatients complained of gross hematuria after ureteral stent removal. Urosepsis occurred in one case after PCNL for an infected renal stone. The rate of complications did not substantially differ from the standard technique using cystoscopy. For the removal in outpatient (12 stents), no major or minor complications were observed.

The mean VAPS was 1.4 for stent removal using a crochet hook, and 2.4 for stent removal using cystoscopy. (p = 0.02) (Table 3)

**Table 3:** Patients Characteristics and clinical outcome in outpatient clinic.

Variables	Number (%) or Median (mean ± SD)		*P*
	CH Removal	STD Removal	
No. of Stents	12	10	
No. of Pts.	12	10	
Age (yr)	64.5 (63.3±14.6)	53.9 (52.9±16.4)	n.s.
Side			
Right (%)	5 (41.7%)	5 (50.0%)	n.s.
Left (%)	7 (58.3%)	5 (50.0%)	
Indication for stenting			
Stone disease (%)	12 (100%)	12 (100%)	n.s.
Anesthesia/Sedation/Pain Killer			
Diclofenac sodium (50 mg)	12 (100%)	10 (100%)	n.s.
Success of ureteral stent removal			
VAPS	1 (1.4±0.7)	2 (2.4±0.8)	0.02

VAPS: 5 grades visual analogue pain scale.

## Discussion

Ureteral stents were first developed in 1967. Since then, various materials and coatings have been developed to avoid ureteral stent complications such as encrustation, incrustation and infections [10], [16]. The incidence of encrustation increases with the duration that the stent remains indwelling [5], [10]. Therefore, stents require periodic replacement or removal.

The standard technique used to remove a ureteral stent is under cystoscopy. A grasping forceps or myocardial biopsy forceps has been used occasionally for the removal or exchange of ureteral stents under fluoroscopy [14], [17]. Various other techniques have been reported for ureteral stent removal using fluoroscopy. A ureteral stent removal procedure without fluoroscopy or cystoscopy was reported by Taylor *et al.* [18]. However, that reported procedure required a special ureteral stent with a magnet. We herein report a simple procedure for ureteral stent removal without fluoroscopy or cystoscopy in female patients that does not require any special type of stent.

The crochet hook is used for knitting in order to make fabric and knit patterns from yarn, such as sweaters. The crochet hook comes in many sizes and materials, and there are a variety of hook sizes. (Fig. 1a) We mainly used a No. 4.5 hook made of metal, which is the same diameter as a 7.5Fr catheter.

A total of 49 of 54 stents excluding migrated stents were successfully grasped, although five stents could not be grasped. Stents that could not be grasped without fluoroscopy were removed under a fluoroscopy-assisted procedure using either a crochet hook or cystoscopy. The success rate was significantly higher in the good position group in comparison to the poor position group. (Fig. 3) The procedure might be technically advantageous in that the bladder does need to have any residential urine to allow the crochet hook to grasp the distal end of the stent easily. Technical success was easily obtained after three or four experiences by three different surgeons, thus indicating the simplicity of the procedure.

This procedure may be useful in the field of clinical urology because this procedure does not require fluoroscopy or cystoscopy. Only one case required an additional procedure using fluoroscopy in the current series, and four cases required cystoscopy for stent removal. Therefore, cystoscopy or fluoroscopy should be prepared before the procedure in case the ureteral stent cannot be removed using the crochet hook. Our complication rate was low, including urosepsis following PCNL with infected renal stones in one patient. However, this urosepsis may have been secondary to PCNL with infected renal stones. There was no active bleeding from the bladder mucosa after ureteral stent removal using the crochet hook technique after URS and PCNL. Gross hematuria was seen in almost all patients, but it was likely the result of ureteroscopic procedures. The rate and grade of hematuria was not significantly different than that with URS and none of the outpatients experienced gross hematuria after ureteral stent removal using a crochet hook.

This removal procedure may be more tolerable than cystoscopy assisted removal. The VAPS of stent removal was significantly lower using a crochet hook than that observed with stent removal using cystoscopy. This may be due to the small diameter of the hook in comparison to the cystoscope (22.5Fr).

This procedure has the advantage of not requiring the use of cystoscopy. Chang *et al.* reported that ureteral stent exchange under fluoroscopic guidance without cystoscopy reduces the cost by about 100USD compared to when the procedure is performed with both fluoroscopic and cystoscopic guidance [11]. According to the Japanese insurance system, ureteral stents removal using cystoscopy costs 10,000 JPY (about 128 USD).

A major limitation of this study was that the successful rate using crochet hook was lower than that for cystoscopy. The main benefit of the crochet technique is that it is easy to perform, requires no cystoscopy or fluoroscopy is cost effective, and finally is less painful. Further studies are needed to confirm the efficiency of this procedure by comparing the benefits and the success rates among the various procedures.

In conclusion, ureteral stent removal using a crochet hook is easy and safe to perform. This procedure does not require fluoroscopy or cystoscopy. This technique was easily acquired and is suitable for use on an outpatient basis. The results of our study showed that the removal of a ureteral stent using the crochet hook technique is usually well tolerated with minimal complications.

## References

[1] Bultitude MF, Tiptaft RC, Glass JM, Dasgupta P (2003). Management of encrusted ureteral stents impacted in upper tract.. Urology.

[2] Borboroglu PG, Kane CJ (2000). Current management of severely encrusted ureteral stents with a large associated stone burden.. The Journal of urology.

[3] Mohan-Pillai K, Keeley FX, Moussa SA, Smith G, Tolley DA (1999). Endourological management of severely encrusted ureteral stents.. Journal of endourology/Endourological Society.

[4] Schulze KA, Wettlaufer JN, Oldani G (1985). Encrustation and stone formation: complication of indwelling ureteral stents.. Urology.

[5] el-Faqih SR, Shamsuddin AB, Chakrabarti A, Atassi R, Kardar AH (1991). Polyurethane internal ureteral stents in treatment of stone patients: morbidity related to indwelling times.. The Journal of urology.

[6] Bukkapatnam R, Seigne J, Helal M (2003). 1-step removal of encrusted retained ureteral stents.. The Journal of urology.

[7] Singh I, Gupta NP, Hemal AK, Aron M, Seth A (2001). Severely encrusted polyurethane ureteral stents: management and analysis of potential risk factors.. Urology.

[8] Somers WJ (1996). Management of forgotten or retained indwelling ureteral stents.. Urology.

[9] Xu C, Tang H, Gao X, Yang B, Sun Y (2009). Management of forgotten ureteral stents with holmium laser.. Lasers in medical science.

[10] Kawahara T, Ito H, Terao H, Yoshida M, Matsuzaki J (2011). Ureteral Stent Encrustation, Incrustation, and Coloring: Morbidity Related to Indwelling Times.. J Endourol.

[11] Chang RS, Liang HL, Huang JS, Wang PC, Chen MC (2008). Fluoroscopic guidance of retrograde exchange of ureteral stents in women.. AJR American journal of roentgenology.

[12] Kim BM, Park SI (2008). Placement of double-J ureteric stent using the pull-through technique in patients with tight ureteric stenosis.. Abdominal imaging.

[13] Ozkan O, Akinci D, Bozlar U, Ustunsoz B, Ozmen M (2009). Retrograde ureteral stent exchange under fluoroscopic guidance.. Diagnostic and interventional radiology.

[14] Park SW, Cha IH, Hong SJ, Yi JG, Jeon HJ (2007). Fluoroscopy-guided transurethral removal and exchange of ureteral stents in female patients: technical notes.. Journal of vascular and interventional radiology: JVIR.

[15] Ho CH, Huang KH, Chen SC, Pu YS, Liu SP (2009). Choosing the ideal length of a double-pigtail ureteral stent according to body height: study based on a Chinese population.. Urologia internationalis.

[16] Zimskind PD, Fetter TR, Wilkerson JL (1967). Clinical use of long-term indwelling silicone rubber ureteral splints inserted cystoscopically.. The Journal of urology.

[17] de Baere T, Denys A, Pappas P, Challier E, Roche A (1994). Ureteral stents: exchange under fluoroscopic control as an effective alternative to cystoscopy.. Radiology.

[18] Taylor WN, McDougall IT (2002). Minimally invasive ureteral stent retrieval.. The Journal of urology.

